# The challenges of educational change: cultural and psychological inertia

**Published:** 2013-09-30

**Authors:** Marcel D’Eon

**Affiliations:** University of Saskatchewan, Saskatoon, Saskatchewan, Canada

In this issue of the CMEJ we showcase several studies of successful innovations, a commentary calling for more and better research into curriculum delivery models to eventually improve the choices available to curriculum planners, and a suggestion that a recent change in resident work hours was ill advised. Applied research, which is much of what currently happens in medical education, by its very nature, recommends, suggests, and offers a new direction which then implies change for someone. On a regular basis we seem to blithely toss out ideas for improvement with little regard for the very real challenges of successfully adopting, implementing, and embedding^1^ new ways into often reluctant organizations. Though we know much about the march of progress as a whole^1^ we know little and use less of what is going on inside individuals and organizations.

Though volumes of books and stacks of articles offer advice, plans, and promises of easy and successful change,^2^,^3^,^4^ the fact remains that significant change remains elusive. Some might argue that significant, persistent, and widespread change has not happened in medical education since Flexner’s influential report in 1910. And even when we are successful, we are not sure why. Some of the latest research has shown that leaders, who are by definition change agents, come in all shapes and sizes and seem to exhibit all manner of characteristics. We still do not really know what makes a leader effective^4^,^5^ in the face of unavoidable and powerful resistance.

From where does the all-too-familiar resistance originate? What is it that makes organizations reluctant? The answer to both questions is people. We are the enemy! All of us at different times and in different ways provide resistance and balk at the prospect of change. Are we bad people? No, we are like everyone else who is empowered and at the same time imprisoned by organizational culture and a psychology of inertia. When change comes knocking we realize, sometimes unconsciously, that we are about to face loss, awkwardness, incompetence, uncertainty, confusion, and conflict.^5^ Under those perceptions of reality it is quite reasonable to resist.

Most new ideas represent a threat to old, familiar, and comfortable ways of being at work. Those that do not pose a threat are by definition not that new, at least not to those of us who embrace them, advocate for them, and attempt to implement them. Researchers and authors in this edition and in general have thought about these new ideas, tried them out, made careful observations, written up their results, and now invite us too to give them a whirl. They have come to terms with the implications of the change, become competent in other ways, make sense of the innovation, and moved forward. Marris^6^ said it well: “…reformers have already assimilated these changes to their purposes, and worked out a reformulation which makes sense to them, perhaps through *months or years* (my emphasis) of analysis and debate.” 6,7

Many of the rest of us are still stuck in our usually helpful but always restrictive organizational culture. It will take time for the rest of us to come around, lots of time. To highlight the startling fact that these ideas are not new I quote again from Marris, who wrote in 1975: “No one can resolve the crisis of reintegration on behalf of another. Every attempt to pre-empt conflict, argument, protest by rational planning, can only be abortive: however reasonable the proposed changes, the process of implementing them must still allow the impulse of rejections to play itself out.” 6,7

Unfortunately, leaders and other change agents become impatient and overly eager for the change. They make decisions, adopt unpopular changes, compromise, and tell their followers all the important reasons why they need to get with the program. And it does not work. Forced change is superficial or it is sabotaged or both and therefore almost always counterproductive.

Real change needs to go deeper than and beyond mere policies and procedures and organizational charts and new course names. Real change penetrates cultural beliefs and assumptions to assimilate or displace the old.^8^ The new way then becomes the way of doing things and the unspoken but pervasive value upon which the organization thrives and is then inevitably constrained.

Take for example changes in undergraduate curriculum and negotiating the proportion of time (and hence priority) that various courses, competencies, or blocks will have. The allocation of time and weight given to public and preventive medicine, knowledge and skills of patient advocacy, inter-professional practice and collaboration, leadership, and of course to clinical decision-making and the scientific basis for medicine merely represent the outcomes of deep cultural assumptions about what really matters and what is truly important. Though stakeholders may generally have espoused beliefs represented even by officially sanctioned and heralded documents like The Future of Medical Education in Canada^9^ or the CanMEDs^10^ roles or by their own statements of educational philosophy, it is the deeply held and shared cultural assumptions and warrants that will carry the day, day after day. These may become clear and even publically uttered during intense negotiations for curricular time when people make statements like, “We all know that the basic sciences have to be mastered first!” These beliefs can trump even FMEC and may command that the petals of the CanMEDs roles be small and withered. Clearly, as Goldsmith puts it: “culture eats strategy for breakfast!” 10

Is there hope for change? Yes, but we must go slowly and not ignore human nature. Being unrealistic, trying to go too far too fast is, sadly, as I stated earlier, truly and predictably counterproductive. We must marry reach with realism as Evans^5^ wrote. We need to find that balance between too fast and too slow, between fierce resistance and suffocating stagnation. Setting, working towards, and then achieving moderate goals will actually give us small wins to celebrate and motivate us to strive for more and better. On the other sad hand, aiming too high too soon leads to burnout and demoralization. Our leaders and change agents, including researchers with great ideas, need to listen, acknowledge the real challenges of change, adopt a much longer time frame and longer horizon, and celebrate the small but important steps that will eventually lead both psychologically and practically to bigger and better. In this spirit let’s explore all those promising ideas for medical education out there, many of them no farther away than this issue of the CMEJ.

Bishop et al. evaluated the use of structured opportunities to better train family medicine residents to quickly find, evaluate, and plan to incorporate into practice, the answers to their own clinical questions with promising initial results.

Veras et al. assessed the level of knowledge and skills in global health in family medicine residents in five universities across Ontario. They found, not unexpectedly, areas of strength and weakness. This can be used to help guide program development.

Using questionnaires, Persson et al. examined attitudes of parents of patients towards medical students learning in pediatric settings. The results could easily be used to strengthen the undergraduate curriculum and teaching in pediatric ambulatory clinics. Interestingly, students rated their skills in communication, history taking and physical exam lower than did the parents.

Using a prospective, cross-sectional study with a national survey of second-year family medicine residents, Janke et al. explored the relationship between sleep deprivation and fatigue among residents and motor vehicle crashes. They found that a higher percentage of residents in rural areas reported adverse motor vehicle events than those working in urban areas. They conclude with a call to action, the need for which is supported by these data.

Wijerathne and Rathnayake used computer-assisted spot tests with medical students in Sri Lanka and found they were well received by the medical students.

Ting et al. explored the Health Care Team Challenge. While they note that the HCTC is resource-intensive they recommend both: (1) that other inter-professional education activities use some of the features of the HCTC, and (2) that the use of the HCTC itself should be expanded to include more students, more teams and more institutions. It seems they can’t get enough of a good thing!

D’Eon makes a case for grand curriculum studies in real medical schools especially in the near future when innovative curricula will allow us to more clearly determine what is working and why. This could free curriculum planners to combine researched features of classical curriculum designs in creative ways.

Razik and Slessarev examined resident work-hour restrictions from a Canadian perspective and delineate some of the reasons why changes to the current call structure may have potentially deleterious effects to all those concerned. They advise us against a top-down approach to change based on erroneous cultural assumptions that we can make things better within the highly complex and interdependent world of hospital medicine by simply imposing, however well intentioned, our pet innovations. They conclude with a plea that all stakeholders consider the potential unintended consequences of a change adopted with the best of intentions. Their caution towards change is different than mine but connected and complementary. Together we implore leaders and change agents to move forward more slowly, to consider the resistance more carefully, to heed contraindications, and to be vigilant for adverse side effects.
